# Antioxidants as Modulators of NETosis: Mechanisms, Evidence, and Therapeutic Potential

**DOI:** 10.3390/ijms26115272

**Published:** 2025-05-30

**Authors:** Fabiola Zambrano, Pamela Uribe, Mabel Schulz, Carlos Hermosilla, Anja Taubert, Raúl Sánchez

**Affiliations:** 1 Center of Excellence in Translational Medicine—Scientific and Technological Bioresource Nucleus (CEMT—BIOREN), Faculty of Medicine, Universidad de La Frontera, Temuco 4780000, Chile; fabiola.zambrano@ufrontera.cl (F.Z.); pamela.uribe@ufrontera.cl (P.U.); mabel.schulz@ufrontera.cl (M.S.); 2 Department of Preclinical Sciences, Faculty of Medicine, Universidad de La Frontera, Temuco 4780000, Chile; 3 Department of Internal Medicine, Faculty of Medicine, Universidad de La Frontera, Temuco 4780000, Chile; 4 Institute of Parasitology, Justus Liebig University Giessen, 35392 Giessen, Germany; carlos.r.hermosilla@vetmed.uni-giessen.de (C.H.); anja.taubert@vetmed.uni-giessen.de (A.T.)

**Keywords:** NETosis, antioxidants, reactive oxygen species (ROS)

## Abstract

Neutrophil extracellular trap (NET) formation is a process known as NETosis and is a critical innate immune response mechanism that can become pathologically dysregulated in various inflammatory, autoimmune, infectious, and neoplastic diseases. Reactive oxygen species (ROS) play a central role in NETosis induction, making antioxidants a promising therapeutic approach. This review outlines the molecular mechanisms underlying NET formation and highlights three principal antioxidant-based inhibitory strategies: NADPH oxidase (NOX) inhibition, ROS scavenging, and myeloperoxidase (MPO) inhibition. Evidence supports the use of agents such as diphenylene iodonium (NOX inhibitor), N-acetylcysteine and glutathione (ROS scavengers), and thiocyanate (MPO inhibitor), which significantly reduce NETosis in vitro and in vivo. Moreover, natural compounds like resveratrol show pleiotropic effects by modulating neutrophil activation, ROS production, and protease activity. Combination therapies that enhance total antioxidant capacity are particularly effective, though their translation to clinical practice faces challenges such as stimulus specificity, bioavailability, and maintaining immune competence. Antioxidant-based therapies thus represent a promising avenue for targeted NETosis modulation. Future research should focus on improving delivery systems, identifying NET-specific biomarkers, and integrating antioxidants into broader immunomodulatory strategies.

## 1. Introduction

NETosis is a specialized form of cell death to neutrophils, a type of white blood cell crucial for innate immunity. During NETosis, neutrophils release web-like structures known as neutrophil extracellular traps (NETs), composed of DNA, histones, and antimicrobial proteins, to capture and neutralize pathogens [1]. While NETosis plays a vital role in host defense, it can also contribute to inflammation and tissue damage, making it a double-edged sword in immune responses [2].

NETosis can occur through different pathways, primarily categorized into “suicidal” and “vital” NETosis, depending on whether the process results in cell death [3,4]. The formation of NETs is heavily dependent on reactive oxygen species (ROS), which can be generated through NADPH oxidase (NOX) or mitochondrial pathways [5]. Autophagy, a cellular degradation process, also plays a role in regulating NETosis, influencing both pathogen clearance and inflammation [6,7].

NETs are effective in capturing and killing a wide range of pathogens, including bacteria, viruses, fungi, and protozoa, thereby preventing their spread within host tissues [2,8]. In the context of viral infections, NETs contribute to antiviral immunity but can also lead to immunopathology if not properly regulated [9]. In teleost fish, NETosis has been shown to be crucial for bacterial defense, highlighting its evolutionary conservation as an immune mechanism [7].

**Figure 1:**
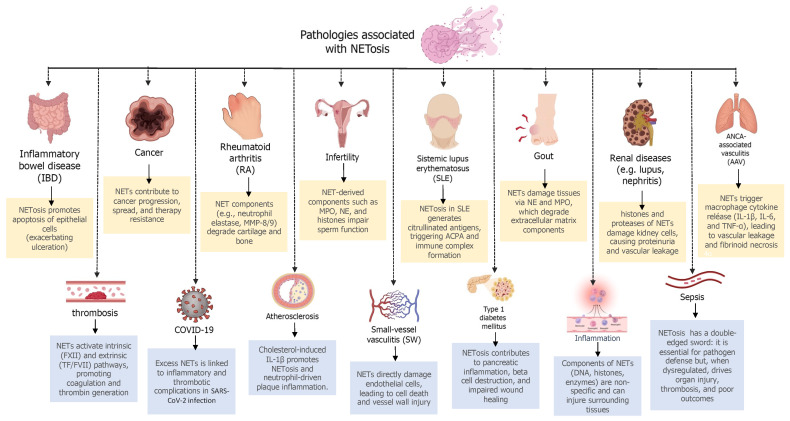
Pathologies associated with NETosis. Schematic overview of major human diseases in which neutrophil extracellular traps (NETs) contribute to pathogenesis through mechanisms such as tissue damage, immune activation, thrombosis, and impaired healing. Examples include autoimmune, infectious, inflammatory, metabolic, and reproductive disorders. Created with BioRender.com.

While NETosis is essential for pathogen defense, excessive or dysregulated NET formation is implicated in various diseases (Figure 1). NETs can contribute to the pathogenesis of conditions such as inflammatory bowel disease (IBD) [10], cancer [11], rheumatoid arthritis (RA) [12], infertility [13,14], systemic lupus erythematosus (SLE) [15], gout [16], renal diseases [17], ANCA-associated vasculitis [18], thrombosis [19], COVID-19 [20], atherosclerosis [21], small vessel vasculitis [22], type I diabetes [23], and sepsis [3], among many others, by promoting inflammation and tissue damage [3,13].

This review aims to explore how antioxidants inhibit NETosis, focusing on their mechanisms of action and antioxidant therapeutic potential. We summarize current evidence and highlight future research directions in oxidative stress related to NETs.

## 2. Mechanisms of ROS in NETosis Activation

ROS play a crucial role in the regulation of NETosis by inducing DNA damage and repair mechanisms. ROS can be generated through different sources.

### 2.1. Via NOX2 or Mitochondrial Activity

NOX2-dependent NETosis is initiated by stimuli such as phorbol esters (e.g., PMA), pathogens, or immune complexes, which activate signaling pathways (e.g., PKC or RAF-MEK-ERK) that trigger NOX assembly. NOX generates superoxide (O_2_^−^), which is converted to hydrogen peroxide (H_2_O_2_) by superoxide dismutase (SOD) [24,25]. H_2_O_2_ fuels MPO-mediated hypochlorous acid (HOCl) production, amplifying oxidative stress and causing DNA damage [26]. This recruits DNA repair proteins (e.g., APE1, PARP1) that inadvertently decondense chromatin by disrupting histone–DNA interactions, aided by NE-mediated histone cleavage and PAD4-driven citrullination [24,27]. Gasdermin D (GSDMD), a pore-forming encoded protein, forms plasma membrane pores, enabling extracellular release of chromatin decorated with antimicrobial proteins. Inhibitors like diphenylene iodonium (DPI) block NOX2, while antioxidants (e.g., catalase) scavenge H_2_O_2_, suppressing this ROS-driven suicidal NETosis pathway [3,28,29].

### 2.2. Via NOX-Independent NETosis

NOX-independent NETosis driven mtROS is activated by calcium ionophores (e.g., A23187), sterile injury, or UV radiation, which elevate intracellular Ca^2+^, triggering mitochondrial calcium uniporter (MCU) and SK3 channel activity to disrupt mitochondrial membrane potential and generate mtROS via a complex III-derived superoxide (converted to H_2_O_2_) [25,30]. This mtROS induces oxidative DNA damage, recruiting base excision repair (BER) proteins (e.g., APE1, PARP1) that decondense chromatin, while bypassing NOX2 entirely [5,25]. Unlike NOX-dependent pathways, this process is faster (~1 h), highly calcium-dependent, and suppressed by mitochondrial uncouplers (e.g., FCCP) or mtROS scavengers like MitoTEMPO [5,28,30]. It plays a role in sterile inflammation (e.g., systemic lupus erythematosus) and offers therapeutic targets such as MCU/SK3 inhibitors to mitigate NETosis without impairing antimicrobial defenses [30].

### 2.3. Via MPO Oxidation Pathway

The MPO oxidation pathway is critical for NETosis. MPO catalyzes the conversion of hydrogen peroxide (H_2_O_2_) to HOCl, which amplifies oxidative stress and induces DNA damage, a prerequisite for chromatin decondensation [31]. During PMA-induced NETosis, MPO cooperates with NE, and thereafter NE translocates to the nucleus to cleave histones, while MPO facilitates chromatin decondensation via ROS-mediated pathways. Pharmacological inhibition or genetic deficiency of MPO significantly reduces NET formation under these conditions, confirming its essential role [31,32]. However, MPO is dispensable in certain bacterial-induced NETosis (e.g., *Pseudomonas aeruginosa*), highlighting stimulus-specific mechanisms [31]. MPO also forms a redox-active complex with NE, where NADPH oxidase-derived ROS triggers NE release into the cytosol. NE then degrades F-actin to halt cytoskeletal dynamics and relocates to the nucleus, enabling DNA release [33]. MPO deficiency alters phosphatidylserine exposure and autophagy-related markers (e.g., LC3-II and p62), though without clear autophagy activation, suggesting that MPO’s role extends beyond oxidative killing to regulating cell death pathways during NETosis [32]. Thus, MPO-driven HOCl production and NE activation are pivotal in oxidative chromatin remodeling, particularly in NOX-dependent NETosis.

The mechanisms underlying NETosis, whether driven by NOX2, mitochondrial ROS, or MPO, converge on ROS-induced chromatin decondensation and extracellular release. While tightly regulated under homeostatic conditions, dysregulated NET formation contributes to the pathogenesis of numerous diseases. The stimulus-specific nature of NETosis highlights the need for targeted interventions that modulate distinct oxidative pathways. Given this, therapeutic strategies aiming to fine-tune rather than completely suppress NET formation have gained traction. The next section discusses emerging therapies that inhibit NETosis in a context-dependent manner, with evidence supporting their efficacy across autoimmune, infectious, and neoplastic diseases.

## 3. Evidence and Therapeutic Applications of NETosis Inhibition

NETosis dysregulation contributes to a wide spectrum of pathologies ranging from autoimmune diseases to cancer progression (Figure 1). In autoimmune and inflammatory diseases, several strategies targeting NETosis have demonstrated therapeutic promise. Therapeutic total anti-citrullinated protein antibodies (tACPAs) selectively bind citrullinated histones H2A and H4, effectively reducing NET formation in models of RA, IBD, and sepsis [34,35]. Beyond inhibiting NETosis, tACPAs promote NET clearance by macrophages, underscoring their dual anti-inflammatory and pro-resolving effects [35]. This targeted approach offers a potentially disease-modifying immunotherapy with minimal systemic immunosuppression. Similarly, inhibition of protein arginine deiminase 4 (PAD4), a key enzyme mediating histone citrullination, is effective in models of SLE and septic shock [36]. PAD4 inhibitors like Cl-amidine, BB-Cl-amidine, and GSK484 not only suppress NET formation but also attenuate ovarian cancer metastasis, pointing to PAD4 as a central player in NET-driven pathologies [37]. Complementing these strategies, DNase I-based therapies degrade the extracellular DNA backbone of NETs, alleviating synovial inflammation and enhancing the efficacy of anti-PD-1 immunotherapy in colorectal cancer [38]. Notably, nanoparticle-encapsulated DNase I offers targeted delivery, preserving immune function while significantly reducing breast cancer lung metastases [39]. In acne vulgaris, *Propionibacterium acnes *induces NET formation, contributing to cutaneous inflammation. Inhibition of the NLRP3 inflammasome effectively reduces NETosis in murine models, while MPO inhibitors such as thiocyanate and selenocyanate prevent NET-mediated tissue injury [40,41,42]. These findings highlight the potential of oxidative stress modulators in treating inflammatory skin disorders and reposition dermatological therapeutics as NET-targeting agents.

In the context of cancer therapy resistance, NETs can physically entrap chemotherapeutic agents such as doxorubicin, reducing their availability and efficacy in the tumor microenvironment [43]. This ‘physical resistance’ mechanism has been observed in models of multiple myeloma and bladder cancer, where DNase I treatment restores drug penetration and enhances therapeutic response [39,43]. Similarly, NETs form a barrier against ionizing radiation and cytotoxic immune cells, shielding tumor cells from both radiotherapy and immunotherapy [37,39]. Inhibitors of NE, including BAY 85-8501, as well as DNase I, have been shown to disrupt these protective networks, thereby sensitizing tumors to radiation and enhancing the efficacy of immune checkpoint inhibitors targeting PD-1 and CTLA-4 in colorectal and pancreatic cancers [38].

Among the diverse strategies targeting NETosis, antioxidant compounds have gained particular interest due to their ability to modulate ROS-dependent pathways central to NET formation. The following section explores the mechanistic basis and therapeutic potential of antioxidants as NETosis inhibitors across various pathological contexts.

## 4. Antioxidants as NETosis Inhibitors

NETosis plays a dual role in host defense and pathological inflammation, driving interest in antioxidant strategies to modulate this process. Research highlights several enzymatic and non-enzymatic antioxidants that effectively inhibit NETosis through diverse mechanisms, particularly by targeting ROS and associated pathways [44,45]. Although direct studies on enzymes like superoxide dismutase (SOD) and catalase are limited, it is plausible to assume that their natural function in neutralizing superoxide and hydrogen peroxide contributes significantly to maintaining redox balance and preventing excessive NET release [28]. The inhibition of NOX by compounds such as DPI and metformin suggests that reducing ROS at the source can effectively disrupt the NETosis cascade [46]. Similarly, the inhibition of MPO by thiocyanate and selenocyanate not only prevents HOCl formation but may also attenuate the downstream tissue damage associated with chronic inflammation [45]. Natural antioxidant like resveratrol has shown multifaceted effects, suppressing MPO and elastase activity, modulating ROS levels, and altering neutrophil behavior, which indicates their therapeutic potential beyond general antioxidative functions [47]. This antioxidant inhibits NETosis through multiple molecular mechanisms that converge on key signaling pathways. Resveratrol has been shown to suppress the activation of nuclear factor kappa-light-chain-enhancer of activated B cells (NF-κB), a central regulator of inflammatory gene expression [48]. By inhibiting NF-κB signaling, resveratrol reduces the transcription of pro-inflammatory cytokines and chemokines that contribute to neutrophil priming and NET formation. In addition, they attenuate ROS production. Furthermore, this polyphenol interferes with upstream kinase cascades involved in NETosis initiation, notably the PI3K (phosphoinositide 3-kinase) and MAPK (mitogen-activated protein kinase) pathways. Through the modulation of these signaling routes, resveratrol effectively dampens neutrophil activation and impair the downstream processes required for chromatin decondensation and extracellular trap release [49]. The synergistic actions of thiol-based compounds, like NAC with glutathione, and vitamins C and E in abolishing NET formation further support the idea that restoring cellular redox homeostasis could be a central strategy to control pathological NETosis [44]. Overall, while more targeted studies are needed, the current evidence supports the conjecture that antioxidant-based therapies may represent a promising avenue for mitigating NET-associated inflammatory tissue damage. Table 1 provides a summary of the main compound with antioxidants activity investigated for their inhibitory effects on NETosis, detailing their mechanisms of action and impact on the process.

NETosis inhibition is achieved by compounds with antioxidant activity, with three main molecular targets offering distinct yet complementary strategies: NOX inhibition, ROS scavenging, and MPO inhibition [44,45]. The NOX inhibition pathway targets the enzymatic production of superoxide (O_2_^−^), a key driver of NET formation in many stimuli. Inhibitors like DPI robustly block both spontaneous and PMA-induced NETosis by halting superoxide generation [45]. Similarly, apocynin disrupts NOX complex assembly, reducing NETs particularly in infectious parasite models such as *Leishmania* [60]. While effective, this approach carries a major caveat: broad NOX inhibition can compromise host antimicrobial defenses, potentially increasing infection susceptibility [60]. On the other hand, ROS scavenging neutralizes excessive ROS production, restoring intracellular redox balance and preventing NET-associated tissue injury. Molecules like N-acetylcysteine (NAC) and glutathione (GSH) reduce NETosis, mitigating reactive nitrogen species (RNS) levels [24,44]. Catalase, which directly scavenges H_2_O_2_—a potent NETosis trigger—shows superior efficacy compared to superoxide dismutase (SOD) [45,61]. Additionally, combined antioxidant therapies (e.g., vitamin C/E + GSH) synergistically enhance suppression of LPS-induced NETs, outperforming monotherapies [44]. Finally, MPO specific inhibition interferes with the downstream formation of HOCl, a cytotoxic oxidant essential for chromatin decondensation in NETosis [45]. Inhibitors such as 4-ABAH and thiocyanate effectively block HOCl production, reducing NET release in PMA- and HOCl-induced settings. However, MPO inhibition exhibits context specificity—being ineffective against NETosis triggered by *Leishmania* or calcium ionophores, which activate MPO-independent pathways [45,60].

**Figure 2:**
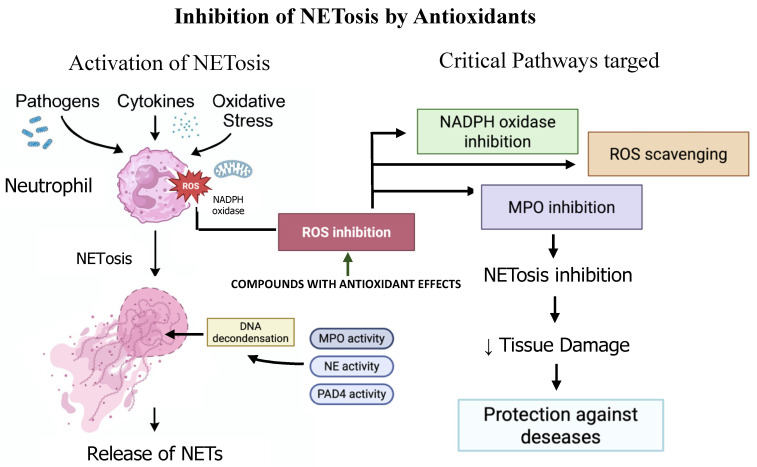
Inhibition of NETosis by antioxidants and the molecular pathways involved. The activation of neutrophil extracellular trap (NET) formation is triggered by microbial pathogens, pro-inflammatory cytokines, and oxidative stress, primarily mediated through NADPH oxidase-derived reactive oxygen species (ROS). This cascade promotes DNA decondensation and the release of NETs, a process facilitated by key enzymes such as myeloperoxidase (MPO), neutrophil elastase (NE), and peptidylarginine deiminase 4 (PAD4). Antioxidants inhibit NETosis by targeting critical signaling pathways, including direct ROS scavenging, inhibition of NADPH oxidase, and suppression of MPO activity. This multi-level interference leads to a reduction in tissue damage and contributes to protection against NET-associated diseases. Created with BioRender.com.

Taken together, these three pathways offer a rational framework for targeted NETosis modulation (Figure 2). NOX inhibitors with MPO blockers show enhanced efficacy, especially in ROS-dependent NETosis [44]. Nevertheless, a critical therapeutic balance must be maintained: while suppression of pathological NETs is desirable, complete NETosis inhibition may impair innate immunity. Thus, future strategies must aim for selective, context-specific modulation to mitigate NET-driven pro-inflammatory pathology without compromising host defense.

## 5. Synergistic Strategies: Antioxidants in Combination Therapies

A recent study highlighted the potential of combinatorial approaches that integrate antioxidants with enzymatic NET-disrupting agents to achieve superior therapeutic outcomes. The concomitant use of GSH and NAC significantly enhanced inhibitory effects on NET formation compared to the administration of either compound alone, by a synergistic interplay in mitigating oxidative stress [44]. While DNase I enzymatically degrades the DNA backbone of pre-formed NETs, antioxidants such as GSH and NAC act upstream by neutralizing ROS, thereby preventing the initiation of NETosis [44]. Furthermore, DNase I primarily attenuates the late-phase oxidative burst, whereas antioxidants target early ROS generation, collectively providing a more comprehensive blockade of NET formation [62]. These findings underscore the therapeutic relevance of combining antioxidants with NET-targeting agents to disrupt both the formation and persistence of NETs in inflammatory pathologies.

In parallel, preclinical studies have demonstrated the therapeutic benefit of combining DNase I with conventional antibiotic regimens. In a murine model of abdominal sepsis, the co-administration of DNase I and antibiotics markedly reduced systemic inflammation, tissue damage, and mortality, outperforming the effects observed with either treatment alone [63]. Notably, spectral analysis revealed a significant reduction in Gram-negative bacterial load following the combined intervention, suggesting enhanced bacterial clearance [63]. Furthermore, this synergistic strategy led to a substantial decrease in both the number of bacterial colony-forming units (CFUs) and overall microbial diversity, with the most pronounced effects observed when antibiotics were administered in conjunction with DNase I [63]. These findings support the concept that dismantling the NET scaffold enhances antimicrobial penetration and efficacy, positioning DNase I-antibiotic combinations as a promising approach to overcome infection persistence associated with NET-mediated bacterial protection.

## 6. Challenges and Future Directions

Although antioxidant-based strategies to modulate NETosis have shown promise in preclinical models, their translation into effective clinical or veterinary applications remains complex. To date, no clinical trials directly assessing antioxidants as NETosis inhibitors in humans have been reported. Available evidence is largely limited to ex vivo studies using isolated human neutrophils and in vivo preclinical models—such as murine models of gout, sepsis, and autoimmune disease. These findings provide strong mechanistic support, but the absence of clinical data underscores the need for translational research efforts, including early-phase human trials, to determine safety, efficacy, and therapeutic relevance in NET-associated pathologies. One of the primary challenges lies in the stimulus-dependent variability of NETosis pathways, where certain triggers induce ROS-dependent mechanisms while others proceed via ROS-independent routes. This mechanistic heterogeneity complicates the selection of universally effective antioxidants. Furthermore, many compounds that demonstrate strong in vitro efficacy often fail in vivo due to poor bioavailability, rapid metabolism, and limited tissue targeting—highlighting that dosing and pharmacokinetics are critical factors for successful translational application.

An additional concern in the use of antioxidants is their long-term use. While antioxidants are vital for neutralizing excess free radicals, long-term supplementation, particularly at pharmacological doses, disrupts immune function, increases infection/cancer risks, and may worsen redox balance. The risk of immune suppression is another concern, given that while excessive NET formation contributes to tissue damage and chronic inflammation, NETs also serve vital antimicrobial functions. Therefore, any therapeutic strategy must strike a delicate balance between dampening pathological NETosis and preserving host defense.

To overcome these limitations, future research should focus on the development of targeted delivery systems (e.g., nanoparticles, prodrugs) that enhance the tissue-specific action of antioxidants while minimizing systemic effects. Equally important is the identification of reliable biomarkers capable of differentiating between physiological and pathological NET formation. These biomarkers could guide the implementation of precision therapies and enable real-time monitoring of treatment efficacy. Finally, large-scale, well-controlled clinical studies are essential to establish safety profiles, therapeutic windows, and long-term outcomes of antioxidant-based NETosis modulation.

## 7. Conclusions

NETosis has emerged as a key immunopathological mechanism involved in a wide range of diseases, including chronic inflammation, infection, cancer, and reproductive dysfunction. Given the central role of oxidative stress in NET formation, antioxidants—both enzymatic and non-enzymatic—have been extensively studied as potential modulators. This review highlighted the mechanisms by which agents such as DPI, NAC, resveratrol, and glutathione interfere with NETosis through ROS scavenging, NOX inhibition, and MPO suppression, leading to reduced tissue damage in various disease models.

Moreover, emerging evidence suggests that combinatorial therapies—integrating antioxidants with agents like DNase I—may offer superior outcomes by targeting both the initiation and persistence phases of NET formation. However, clinical translation continues to face key challenges, including pathway heterogeneity, limited pharmacokinetic stability of antioxidants, and the necessity to maintain innate immune competence.

In summary, antioxidant-based NETosis inhibition holds considerable therapeutic promise, but its clinical application requires further refinement through targeted delivery technologies, biomarker-driven patient stratification, and rigorous translational research. Addressing these challenges will be critical to advancing this strategy from bench to bedside.

## Figures and Tables

**Table 1:** Summary of compounds with antioxidant activity shown to suppress NETosis.

Antioxidant Compound	Disease Models	Clinical Status	Mechanism of Action	Effect on NETosis	Reference
Vitamin C	Non-specific	Preclinical study	Enhances redox balance and scavenges ROS.	Dose-dependent reduction in NET formation; complete prevention at pharmacological concentrations.	[44]
Trolox, Tempol	Non-specific	Preclinical study	Scavenge ROS and inhibit enzymes responsible for ROS synthesis.	Inhibit ROS-dependent NET release.	[50]
Catalase	Pan-inflammation	Preclinical study	Enhances redox balance and scavenges ROS.	Inhibits NETosis via H_2_O_2_ scavenging.	[5,51]
Proanthocyanidin	Liver Cancer	Preclinical study	Significantly reducing ROS levels in neutrophils.	Blocks the oxidative stress pathways required for chromatin decondensation and NET release.	[52]
N-Acetyl Cysteine (NAC)	Non-specific	Preclinical study	Combined with GSH, it abolishes NET formation in LPS-stimulated neutrophils.	Reduces NETs by scavenging ROS and inhibiting MPO activity.	[44,45]
Resveratrol	Non-specific SARS-CoV-2 infection	Preclinical Study Preclinical study	Inhibits myeloperoxidase release, particularly when stimulated with PMA and POVPC, and reduces oxidative stress.	Suppresses DNA release from neutrophils. Decreases the neutrophil-activated status and the release of free DNA, inhibiting NET formation.	[47,49]
Glutathione (GSH)	Non-specific	Preclinical study	Improves intracellular antioxidant capacity and GSH/GSSG ratio.	Strong suppression of NET formation in serum and intracellular levels.	[44]
butylated hydroxytoluene (BHT)	Non-specific	Preclinical study	Enhances redox balance and scavenges ROS.	Reduces NET formation in vitro.	[53]
Metformin	Diabetes	Preclinical and clinical study	Inhibits PKC-NADPH oxidase pathway.	Reduces NET components and blunts NETosis in vitro.	[46]
Thiocyanate, Selenocyanate, Nitroxides	Chronic Inflammation	Preclinical study	Modulate HOCl production by MPO.	Prevent NETosis in PLB-985 neutrophils exposed to PMA and HOCl.	[45]
MitoTEMPO	Chronic obstructive pulmonary disease (COPD)	Preclinical and clinical study	Prevents NADPH oxidase activation.	Reduces NETosis in specific contexts (e.g., COPD mouse model).	[54]
Diphenyleneiodonium (DPI)	Thrombosis	Preclinical and clinical study	Inhibition of NOX2.	Identifies NOX2 inhibition as a potential new therapeutic target for antithrombotic treatment.	[55]
Peroxiredoxin (Prdx)	Inflammatory bowel disease (IBD)	Preclinical study	Upregulation in response to voluntary exercise.	Reduces inflammation and inhibits NETosis in TNBS (induce experimental colitis).	[56]
(+)-Borneol	Non-specific	Preclinical study	Inhibits ROS generation and NADPH oxidase activity.	Decreases ROS levels and inhibits NETosis triggered by PMA stimulation.	[57]
Anthracyclines (e.g., Epirubicin, Daunorubicin)	Non-specific	Preclinical study	Suppress both NADPH oxidase-dependent and -independent NETosis.	Inhibit NETosis without suppressing ROS necessary for antimicrobial functions.	[58]
Indolylmaleimide Derivative IM-93	Non-specific	Preclinical study	Inhibits oxidative stress-induced necrotic cell death.	Inhibits both ferroptosis and NETosis.	[59]
Combination Therapies	Non-specific	Preclinical study	Enhances TAC and reduces ROS/RNS levels.	Most effective approach; achieves strongest suppression of NET formation using Vit E + Vit C + GSH + NAC.	[44]
